# Minimally Invasive Mapping Guided Surgical Treatment of Atrial Fibrillation. Utopia or Near Future?

**Published:** 2006-10-01

**Authors:** Thomas J van Brakel, Gil Bolotin, M.A. Allessie, Jos G Maessen

**Affiliations:** 1 Cardiovascular Research Institute Maastricht, Department of Cardiology; 2 Cardiovascular Research Institute Maastricht, Cardiothoracic Surgery; 3 Cardiovascular Research Institute Maastricht, Department of Physiology; University Hospital Maastricht, The Netherlands

**Keywords:** Atrial fibrillation, Minimal invasive, robot, pulmonary veins, mapping

## Abstract

Isolation of the pulmonary veins has been used as surgical treatment for atrial fibrillation (AF) from the early 90s, as it was incorporated in the Maze procedure. With the evidence that triggers form this area can induce AF, the Maze III procedure has been adapted and modified towards a single lesion around the pulmonary veins for the treatment of paroxysmal and chronic AF in some centers. New ablation techniques with a diversity of energy sources further paved the way for less invasive procedures. Minimal invasive techniques to prevent major surgery may potentially make the treatment available for a patient population that do not have to undergo cardiac surgery for other reasons. Besides these technical developments, high density mapping can be used to identify the AF substrate in the individual patient and optimization of the treatment by local substrate guided ablation. This review aims to summarize the robotic and thoracoscopic techniques to isolate the pulmonary veins. Furthermore, it is discussed why pulmonary veins isolation may be effective in patients with chronic AF, and whether there is a role for mapping guided minimal invasive surgical treatment of AF in the near future.

## Introduction

The Maze procedure was for a long time accepted as the 'gold standard' for the surgical treatment of atrial fibrillation (AF) in a selected group of patients [1]. Although the Maze procedure cured AF in most of the patients (approximately 90%) the technique did not find widespread application due to its complexity and invasiveness. To overcome these drawbacks, the procedure has been adapted and simplified over the last ten years. Techniques such as cryo-, radiofrequency- and microwave ablation have shown to be able to replace the time consuming cut-and-sew technique of the Maze procedure [2,4]. Furthermore, epicardial ablation techniques are currently explored to be able to perform epicardial ablations on the beating heart [2,3,5]. The Maze procedure and modified procedures were originally based on the multiple wavelet hypothesis and aimed to interrupt reentry circuits [6,7]. Interestingly, together with a change in techniques, an important change in theories facilitated the further 'minimalization' of the technique. After Haissaguerre et al demonstrated that ectopic foci from the pulmonary veins can initiate AF [8], surgical treatment of AF concentrated on the pulmonary vein area as the main target area. With the development of minimally invasive techniques, the indication for epicardial pulmonary vein ablation has been widened and allows patients with lone AF to be treated via this approach [5]. Endoscopic and robotic procedures are under investigation and already clinically used in some centers.

Most recently it has become clear from epi- and endocardial mapping studies that areas with fractionated electograms may be responsible for the perpetuation of AF [9]. Since the location of these areas may differ between patients, mapping for substrate identification, followed by substrate targeting, may increase the success percentages of AF treatment significantly. Particularly the epicardial approach offers the advantage that the entire surface of both atria be reached. Furthermore, the myocardium can be treated outside the circulation, decreasing the risk of thromboembolic events and the need for anticoagulants. Although not yet performed, the combination of minimally invasive substrate identification by mapping and targeting with ablation, may be the treatment of choice for the surgical management of AF in the near future.

## Thoracoscopic and Robot-assisted isolation of the pulmonary veins

From a historical point of view, the surgical treatment of AF was only considered appropriate for patients undergoing cardiac surgery for structural heart diseases, or for patients with permanent highly symptomatic AF who are willing to undergo major surgery [10,11].  As long as the procedure remains invasive, the physical and technical limitations make the epicardial approach less interesting as a target for treatment of AF in patients who do not have to undergo cardiac surgery. In an attempt to overcome these issues, surgical techniques are adapted and refined using robotic-endoscopic techniques. Table 1 summarizes the publications on endoscopic or robotic epicardial treatment of AF.

**Figure 1:**
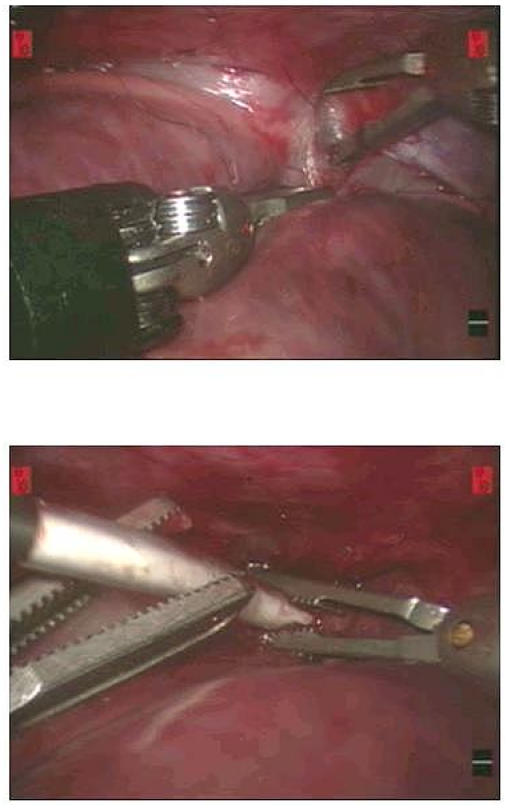
Robot-assited right chest epicardial isolation of the pulmonary veins. The upper panel depicts blunt preparation of the inter-atrial grove. The lower panel shows the introduction of the microwave Flex-10 ablation catheter into the transverse sinus.

Standard, minimally invasive methods using thoracoscopy, are characterized by limited manoeuverability of the instruments with limited two-dimensional vision. The recent developments in robot-assisted surgery may overcome these drawbacks of endoscopic surgery. This approach aims to provide three-dimensional vision techniques and simulation of the surgeons smooth wrist-finger action, through access ports of less than 1 cm [23]. Recently, we reported a safe right sided chest approach with the da Vinci Robotic Surgical System and microwave ablation to isolate the pulmonary veins in dogs (see Figure 1) [14]. Also Smith et al reported a minimally invasive beating heart epicardial left AF procedure using monopolar microwave energy with the da Vinci Robotic Surgical System [13]. Although the robotic system indeed shows a better manoeuvrability of the instruments with better vision, it might not be necessary for this procedure. Several groups have shown fast and safe procedures of epicardial beating heart pulmonary vein ablation with direct thoracoscopic instruments without robotic assistance [19,21,22]. Pruitt et al described a totally endoscopic closed-chest microwave mini-Maze ablation for the treatment of intermittent and continuous AF with 80% of patients free from AF after 8 month of follow up [12]. Also Salenger et al reported a total endoscopic epicardial procedure to isolate the pulmonary veins, with a short length of hospital stay, short procedure time, and acceptable rhythm results [19].

## Why pulmonary vein ablation?

The role of triggering foci originating from the pulmonary veins responsible for the initiation of AF, has been well described [8,24]. Electrical isolation of each pulmonary veins alone, without additional lesions to block pathways of reentry, seems to be quite effective in patients with paroxysmal AF [25,26], although the  results in patients with persistent and permanent AF showed rather disappointing results [4,27]. Gaita et al reported a success percentage of only 25% after complete isolation of the pulmonary vein ostia in patients with permanent AF associated with valvular heart disease [4]. Sanders et al demonstrated that termination of AF occurred during PV ablation in 17 of 19 with paroxysmal and in 0 of 13 patients with permanent AF [28]. Although direct evidence is lacking, these studies indicate that there is a clear difference in mechanisms between paroxysmal and persistent AF. Apparently, the pulmonary veins seem to play an important role in paroxysmal but not in persistent AF.  However, this statement can not be maintained when the posterior wall of the left atrium, which comprises the pulmonary veins, is taken into account. Todd et al showed that the isolated posterior left atrium can sustain AF whereas the remaining much larger atrial tissue could not [29]. They speculated that the anatomy or electrophysiology of the pulmonary vein area may promote reentry, resulting in the maintenance of AF.

Furthermore, surgical experiences have shown that 60-80% of patients with persistent AF can be treated, regardless of the lesion set and the technique used [30]. All these techniques and lesion sets, however, have one thing in common and that is the involvement of the posterior area between the pulmonary veins. The results of these clinical studies are difficult to compare, because of differences in patient groups, use of antiarrhythmic agents and clinical endpoints. What can be derived from these studies is that only isolation of the PV ostia is not enough to cure persistent AF, but that lesions in- or around the posterior left atrium are needed to restore sinus rhythm. Gaita et al randomized patients with persistent AF for pulmonary vein ostial isolation and pulmonary vein ostial isolation in combination with a 7-shaped lesion on the left atrial posterior wall. Interestingly, the low success rate (25%) in the PV group increased to 76% in the 7 lesion group (without antiarrhythmic drugs) [4]. Others also showed that one circular lesion around all pulmonary veins in patients with persistent AF and structural heart disease can restore sinus rhythm 50-80% of patients [3,31].

## Complete or incomplete isolation

It is generally believed that irrespective of the type of device or energy source, ablation lesions need to be transmural to prevent reentry or triggers from initiating or maintaining AF. However, several recent studies showed that in many cases linear ablation lesions around the pulmonary veins are electrically or histologically incomplete [32-34]. Particularly beating heart endoscopic or robotic ablation might increase the risk of incomplete isolation, due to endocardial blood cooling (or in case of cryo energy warming), and difficult anatomic position of the lesions with reduced direct vision of the catheter. In a recent study from our group, complete isolation after a single robot-assisted microwave lesion around the pulmonary veins was demonstrated in 8 out of 16 dogs [15]. In the study of Smith et al it was concluded that further confirmation of catheter positioning is necessary during a right-chest-only robotic approach because lesion transmurality was not demonstrated consistently [13].

Interestingly, most clinical studies of (minimal invasive) surgical isolation of the pulmonary veins for AF treatment, reported high percentages of sinus rhythm restoration [30] whereas it may be assumed that a high percentage of lesions are incomplete. This suggests that more than isolation of triggers from the pulmonary veins alone is needed to treat patients with persistent AF.

## Mapping of the AF substrate as a guide for minimal invasive surgical AF treatment

**Figure 2:**
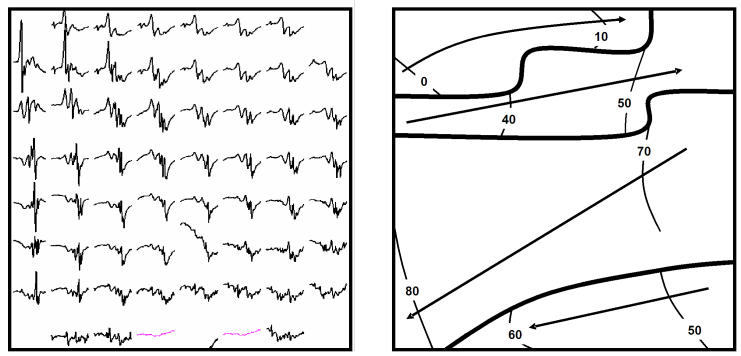
Epicardial high density mapping of the area in between the pulmonary veins. The left panel depicts the 1 cm^2^ 60 unipolar mapping probe signals of a single time window.  On the right side, the wave pattern of this time window is shown. Note the fractionated signals and the small streets of dissociated waves.

AF generally occurs in the presence of an atrial substrate which is the result of structural and/or electrical alterations of the tissue (so called remodeling). Experimental and clinical studies provided insight into these tissue alterations and showed that different types of remodeling are observed in the context of different underlying pathologies (heart failure, rapid atrial activation, valvular disease, hypertension etc). To selectively and locally treat patients with AF, one of the most important and challenging aspects is to study the mechanisms of AF perpetuation in individual patients with different types of atrial remodeling. High density epicardial mapping can give insight into this electropathological substrate. From the mapping data that were recently collected in our clinics, it is clear that focal activity or a rotor acting as a 'driver' with  fibrillatory conduction in the posterior left atrium is not a common mechanism in patients with persistent AF and structural heart disease. Other types of substrates located in the pulmonary vein area need to be considered. Electrical and structural changes of the atria may have progressed into a stage, where a rotor or focal source is no longer necessary for the perpetuation of AF. Nademanee et al also found complex fractionated electrograms mainly confound to the pulmonary veins, the roof of the left atrium and the interatrial septum. Ablation of these fractionated areas resulted in restoration of sinus rhythm in the majority of patients [9]. Interestingly, we did observe a higher percentage of fractionated electrograms and dissociated small waves in the pulmonary vein area and Bachmanns bundle compared to the remaining tissue in the left and right atrium. Figure 2 shows a typical example of fractionated electrograms and small streets of dissociated waves recorded from the area in between the pulmonary veins. As suggested by Shan et al [35], fractionation of electrograms at Bachmann's bundle may be the result of dispersion of refractoriness between Bachmann's bundle and the surrounding tissue, or it may be the expression of local reentry within Bachmann's bundle. Local reentry in Bachmanns Bundle and the pulmonary vein area can act as a perpetuator of fibrillation. Goette et al suggested that in diseased atria, fibrosis and fatty degeneration will cause electrical dissociation of muscle bundles giving rise to "structurally determined" fractionation [36]. If indeed this is a mechanism in patients with persistent AF, ablation of the fractionated areas will contribute to the rate of cardioversion.

Although the role of fractionation and dissociation in the perpetuation of AF is not yet fully elucidated and needs further studies, it might be used as a guide for substrate guided ablation. The hand made mapping probe used in our center has 60 electrodes on 1 cm^2^. With some adaptations, this instrument may be easily transformed for endoscopic or robotic mapping of atrial fibrillation. With online identification of the fractionated and dissociated fibrillation areas, the surgical treatment of AF can shift from an anatomic approach (isolation of the pulmonary veins) to a more rationalized substrate based electrophysiologic approach. By combining mapping, ablation and minimal invasive techniques, rationalized individually tailored surgical treatment of AF may become available for a broader patient population. Whether this new approach will gain a widespread clinical application depends on future developments of the procedure including procedure time, the cost-benefit profile, and the success of treatment.

## Figures and Tables

**Table 1:**
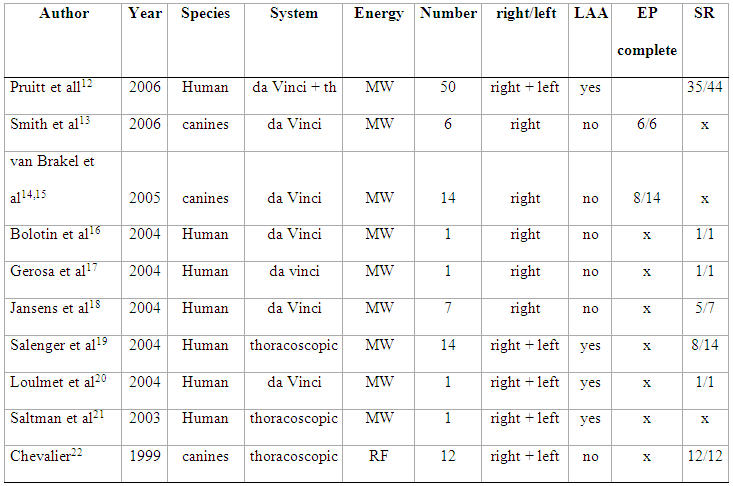
Epicardial robotic/thoracoscopic isolation of the pulmonary veins

MW=microwave, LAA=left atrial appendage, EP=electrophysiology, SR=sinus rhythm
